# Deep Learning and Atlas-Based MRI Segmentation Enable Longitudinal Characterization of Healthy Mouse Brain

**DOI:** 10.3390/jimaging11110418

**Published:** 2025-11-19

**Authors:** Edoardo Micotti, Liviu Soltuzu, Elisa Bianchi, Sebastiano La Ferla, Lorenzo Carnevale, Gianluigi Forloni

**Affiliations:** 1 Department of Neuroscience, Istituto di Ricerche Farmacologiche Mario Negri IRCCS, 20156 Milan, Italy; edoardo.micotti@marionegri.it (E.M.); elisa.bianchi@marionegri.it (E.B.); 2 Unit of Neuro-Cardiovascular Pathophysiology, I.R.C.C.S. Neuromed, 86077 Pozzilli, Italy; 3 Department of Molecular Medicine, Sapienza University of Rome, 00161 Rome, Italy

**Keywords:** neuroimaging, deep learning, mouse, aging

## Abstract

We compared the results of brain magnetic resonance image (MRI) segmentation across a longitudinal dataset spanning mouse adulthood using an atlas-based approach and deep learning. Our results demonstrate that deep learning performs similarly yet faster than more established segmentation methods, even when computational resources are limited. Both methods enabled the large-scale analysis of a cohort of C57Bl6/J healthy mice, revealing sex-dependent morphological differences in the aging brain. These findings highlight the potential use of deep learning for high-throughput, longitudinal neuroimaging studies and underscore the importance of considering sex as a biological variable in preclinical brain research.

## 1. Introduction

The use of neuroimaging technology to evaluate experimental animals is important due to their high translational value, the reduction in the number of animals between groups, and the possibility of following the trajectory of single subjects. Over the last 20 years, many efforts have been devoted to brain segmentation in animal models, which has led to the development of more sophisticated and accurate tools [1]. As in humans, manual brain segmentation remains the “gold standard”; however, it is extremely time-consuming and prone to biases like inter- and intra-operator variability. Thus, researchers have developed atlas-based automatic procedures in which the single-subject brain is extracted from surrounding tissues (skull-stripping) and then co-registered to a segmented atlas through diffeomorphic transformations [2,3]. Atlas-based approaches have led to a significant improvement in speed and repeatability, as well as the generation of common spatial references like the Allen Mouse Brain Atlas [4] and Waxholm Space Atlas [5]; nonetheless, they remain quite slow and require several days to process a typical experiment. With the advent of deep learning (DL) techniques in medical imaging, new algorithms are now in the spotlight for being much faster and, in some cases, more accurate than traditional atlas-based approaches [6]. These new DL approaches have been applied to murine models, addressing three main scientific needs—skull stripping [7,8,9,10,11], parcellation of the intact whole brain [12,13], and segmentation of brains with lesions [14,15,16]—and showing great potential in terms of accuracy and computational time. Here, we have developed a DL-based analysis using nnU-Net, a self-configuring method for medical image segmentation [17], by using a dataset of brain images from mice of different ages, sex, and strains, acquired using varied protocols. Such diversity of subjects is more suitable for comparisons between experimental sets acquired under different conditions. The model proved to be much faster than a multi-atlas (MA) approach and was comparable in terms of accuracy; moreover, the proposed model can be run on personal computers, allowing for widespread use.

Our method has been tested in a longitudinal study of the natural aging in the C57Bl6/J strain, the most common mouse strain used in preclinical research, with particular attention to the comparison between males and females, according to procedures outlined by Italian RIN Neuroimaging Network [18]. Here, we have not only provided a low-cost and easily expandable DL method for brain segmentation, but, for the first time, have also mapped the trajectory of a healthy mouse population stratified by sex at the single-subject level. Our findings show that the mouse brain undergoes significant changes during adulthood and that sexual dimorphism is present in brain regions such as the cortex, striatum, and olfactory bulbs.

## 2. Materials and Methods

We employed two distinct MRI datasets, which are described in detail in the following subsections: the study set (20 animals and 75 MRI volumes) and the training set (161 animals and 161 MRI volumes). The number of animals and MRI volumes included were estimated from previous works with a similar experimental design: for the study set (stratified by sex), see refs. [19,20], and for the training set (including validation), see ref. [13].

### 2.1. Animals

The study set consisted of two sex-balanced groups of C57Bl/6J mice (total *n* = 20) that were followed longitudinally at four time points: 4, 8, 15, and 19 months.

### 2.2. MRI Acquisition Protocol

Animals in the study set were anesthetized using isoflurane in a mixture of O_2_ (30%) and N_2_O (70%). Body temperature was maintained at approximately 37 ± 0.5 °C by a warm-water-circulated heating cradle. MRI experiments were carried out on a Bruker BioSpec 70/30 equipped with Bruker AVANCE III electronics (Bruker, Ettlingen, Germany).

Two actively decoupled radio frequency coils were used: the transmitter was a volume coil of 7.2 cm diameter and the receiver was a surface quadrature coil. A rapid 3D acquisition with relaxation enhancement (RARE) T2-weighted sequence was performed to assess anatomic changes. Morphological images were obtained with a voxel size of 0.1 × 0.1 × 0.1 mm (matrix = 300 × 110 × 80 and field of view = 3 × 1.1 × 0.8 cm) a repetition time TR = 2500 ms, an effective echo time TE = 50 ms, and a RARE factor of 16, for 1 average.

To minimize the potential differences in the spectrometer performance over time, male and female batches were alternated during acquisition. Before further processing, images were visually inspected for quality by an expert researcher; all sets passed inspection.

### 2.3. Multi-Atlas Parcellation

Multi-atlas brain segmentation was performed using a modified version of the pipeline proposed by Pagani et al. [21]. To this end, we created a study-specific multi-atlas template. We selected a homogeneous group of mice from our study set to create a set of brain templates: 10 brain volumes from 4-month-old male subjects. For the following steps, we used scripts from the ANTs [22,23] and FSL [24] toolkits. Single-subject brains were first corrected for the intensity gradient induced by the surface coil (N4BiasFieldCorrection). The ten brains were then co-registered to each other and averaged to obtain a template for the skull stripping process (buildtemplateparallel.sh). The whole-brain mask of the averaged template was manually drawn and back projected to the single subject space (antsIntroduction.sh and WarpImageMultiTransform commands) to perform skull stripping. Once the brains were extracted, a second intensity-gradient correction step was performed to better correct for this phenomenon. After these preliminary operations, brain parcellation was performed using the join label fusion approach (antsJointLabelFusion.sh).

Before accepting the newly created multi-atlas template, a trained researcher checked the results and manually corrected the single-subject segmentations.

Once the new set of 10 reference atlases were ready, the same workflow was applied to the entire test set using the newly created multi-atlas template.

### 2.4. Deep Learning Parcellation

Our training set consisted of 161 mouse brains from different strains (C57BL/6J, APP/PS1, SAMP8, P301S, JNPL3, SAMP8, and SAMR1), sexes (117 males and 44 females), ages (40 four-month-old, 21 six-month-old, 36 eight-month-old, 48 ten-month-old, 14 thirteen-month-old, and 2 sixteen-month-old mice), and acquisition sequences (T1-, T2- and T2*-weighted scans), acquired at the Mario Negri Institute (141 brains) and Neuromed (20 brains). All these data were pooled from previous experiments performed at our institutes.

Each volume was initially segmented using a published mouse brain atlas and the multi-atlas technique. An ex vivo atlas with 114 structures was chosen for its resolution and number of labels [25]. These structures were then grouped into nine major regions (Supplementary Table S1): “amygdala”, “cerebellum”, “cortex”, “hippocampus”, “olfactory bulbs”, “striatum”, “thalamus and hypothalamus”, “ventricles” and “white matter”. A tenth region, called “other”, encompassed all remaining areas. This regrouping was necessary to keep the number of training classes low, yet still useful for a typical MRI experiment. In addition, some brain structures were either too small to be accurately segmented or their boundaries were not clearly detectable at our MRI resolution (e.g., structures from the cortex); thus, an attempt to segment them would have introduced crucial biases. Even though, from a biological point of view, aggregating the regions meant that we were not able to investigate the effect of age on functionally different areas, we decided to regroup using the following empirical criteria: function, relative size and location, and volume homogeneity.

Both original and simplified segmentations were inspected and manually corrected to eliminate large errors, and thus our ground truth data were created; MRI image data were corrected for the intensity gradient using the N4BiasFieldCorrection script. We trained the nnU-Net deep learning network for the supervised multi-class segmentation task using the 3D configuration at full resolution, for 300 epochs; five-fold cross-validation (80% of the data for training, 20% for validation); and the default data augmentation [17]. An additional model was trained using all MRI images in one fold. Unless otherwise specified, all results below were obtained with the five-fold model used as an ensemble, as suggested by the developers of the nnU-Net method. For a detailed network configuration, please consult the original paper.

Training and data analysis were performed using a machine equipped with a 16-core i7 Intel processor, 64 GB of memory and a NVIDIA RTX A4000 GPU. The following software packages were installed on a system running Ubuntu 22.04.5 and an environment with Python 3.9.20, torch 2.5.1, CUDA 11.8, nnU-Net 2.5.1, ANTs 2.5.0, AFNI 23.3.02, and FSL 6.0.7.13.

### 2.5. Statistical Analysis

Statistical analysis was carried out using SAS software (version 9.4, SAS Institute, Cary, NC, USA); all other analyses were performed with custom R and Python code.

The brain volume was calculated for each region and subject for the multi-atlas and deep learning segmentations. Since longitudinal experiments are prone to unbalanced data (death of subjects, data discarded for artifacts), we decided to perform a linear mixed model (LMM) analysis with a random intercept and slope and an unstructured variance-covariance matrix. LMM outputs (intercept and slope) have very precise biological meanings; the intercept models the size of the brain region at the first time point (4 months of age) and the slope models the rate of volume variation with time. When the slope is positive, this indicates an enlargement of the brain region; when it is negative, this indicates that the brain region will shrink with age. Leaving both the intercept and slope as random factors is fundamental to account for inter-individual variability; this is still required even though, unlike humans, laboratory mice are a very homogeneous population.

Our analysis considered age, sex and method (multi-atlas or deep learning) as fixed effects and the subject as a random effect. As brain growth is normal until adulthood, the intercept was set at 4 months of age, at which point mice are considered young adults. Two different models were applied: age*method and age*sex*method. The former is used to evaluate the following aspects: whether there is a change in the mean of single-subject trajectories along time from 4 to 19 months of age (age effect); whether the segmentation method affects the intercept of the linear model (method effect); whether the segmentation method affects the rate of change (slope) of the linear model (interaction between age and method). In the second model, sex was introduced as an additional covariate, along with its interactions with age and method, to check the following: whether sex influences the intercept and/or the slope of the linear model (sex effect; interaction between sex and age); and whether the method affects the intercept and/or slope of the linear model in a sex-dependent manner (interaction between sex and method; interaction between age, sex and method). For a graphical representation of the LMM outputs, see Supplementary Figure S2.

## 3. Results

### 3.1. Computation Time and Model Accuracy

**Figure 1:**
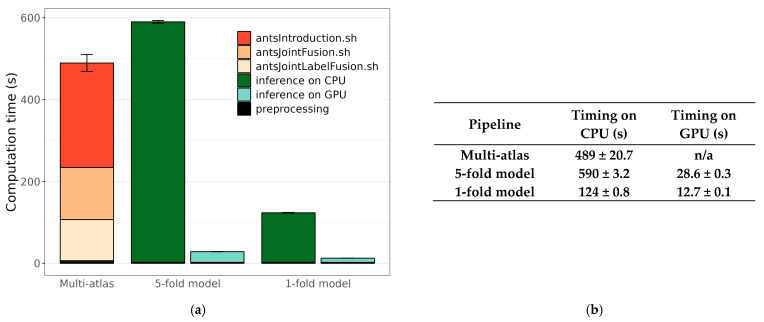
(**a**) Duration of segmentation using the two methods: the multi-atlas technique and deep learning. The colors indicate the different steps of each method. “Preprocessing” includes steps that are too short to be represented (e.g., N4 bias field correction and brain scaling). Error bars indicate ±one standard deviation of the total duration. (**b**) Table reporting total durations and standard deviations of the compared methods.

We measured the duration of the segmentation time of our test MRI brain volumes using two methods: the multi-atlas technique and deep learning (DL) (Figure 1). The multi-atlas pipeline run on average for 489 s; the bulk of this time was taken up by two scripts: antsIntroduction.sh and antsJointFusion.sh (including the wrapper antsJointLabelFusion.sh). The DL pipeline consisted of two steps: N4 bias field correction in the structural MRI image and prediction using the corrected images. Our DL segmentation model was noticeably faster when inference was run on the GPU (28.6 s), yet slower than the multi-atlas technique when run on the CPU (590 s). Considering a scenario in which the user does not have access to a GPU, we trained and timed a model in which all training data was included in a single fold. In this model, inference was almost five times faster on the CPU (124 s) than our main model (and more than two times faster on the GPU, at 12.7 s), making it a competitive choice for personal computers.

**Figure 2:**
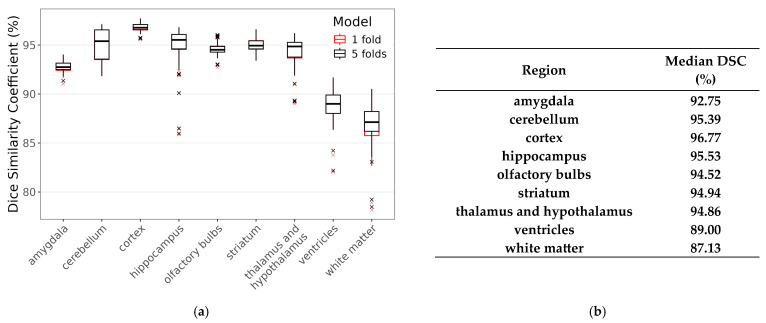
(**a**) Boxplots showing Dice similarity coefficients between the multi-atlas technique and the main model (“5 folds”, in black) and between the multi-atlas technique and the alternative model (“1 fold”, in red). (**b**) Table reporting median Dice similarity coefficients for the “5-fold” model.

We computed the Dice similarity coefficient (DSC) between the multi-atlas segmentation and the prediction of the DL model for each brain and region in our study set. For a given pair of multi-atlas and DL regions, the DSC is calculated as 2*(number of overlapping voxels)/(number of voxels in the first region + number of voxels in the second region). In Figure 2, median DSCs are reported as percentages. DSC% values range from 0 (no overlap) to 100 (perfect overlap), with most regions showing approximately 95% similarity irrespective of the model configuration. We note that the results of our faster model (“1 fold”), suitable for CPU-only computers, are virtually identical to those from the model we used for brain volume estimations (“5 folds”): the total mean difference between DSCs from the two models is 0.0007 and the null hypothesis (i.e., the two models are the same) is supported by the Bayes factor analysis (BF_10_ = 0.06).

### 3.2. Estimation of Brain Region Volume Using Two Segmentation Methods

Longitudinal MRI data are reported as boxplots; outliers have been kept in all statistical analyses. With the aim of clearer data visualization, subjects in graphs have been grouped to the closest age (±0.5 months). For the linear mixed modelling, subjects have been classified using the approximate age (expressed in months) on the day of the scan.

Besides the whole brain, nine regions were considered in this analysis: cortex, hippocampus, striatum, ventricles, olfactory bulbs, amygdala, thalamus and hypothalamus, white matter, and cerebellum.

**Figure 3:**
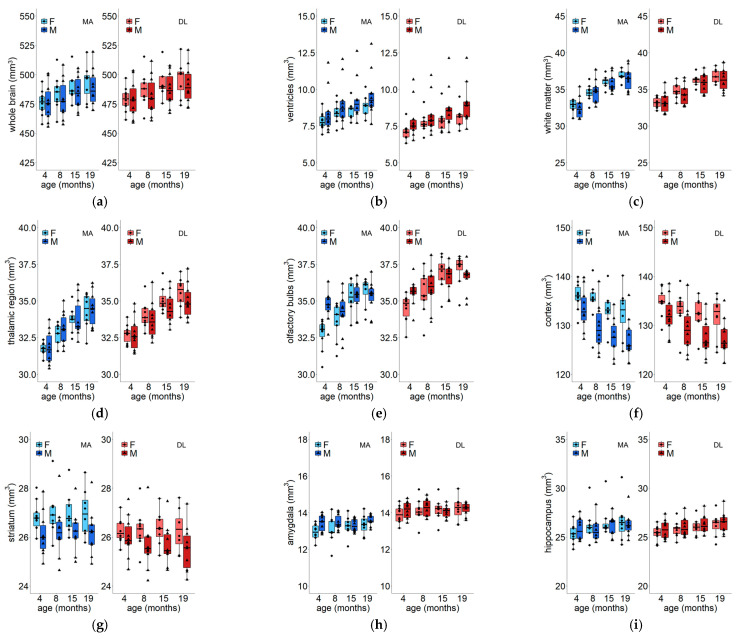
Variation in volume with age for the (**a**) whole brain, (**b**) ventricles, (**c**) white matter, (**d**) thalamus and hypothalamus, (**e**) olfactory bulbs, (**f**) cortex, (**g**) striatum, (**h**) amygdala, (**i**) hippocampus. Dots represent the single-subject values. Color codes: female and multi-atlas (light blue), male and multi-atlas (blue), female and deep learning (light red), and male and deep learning (red). Abbreviations: F = female, M = male, MA = multi-atlas segmentation, DL = deep learning segmentation.

**Figure 4:**
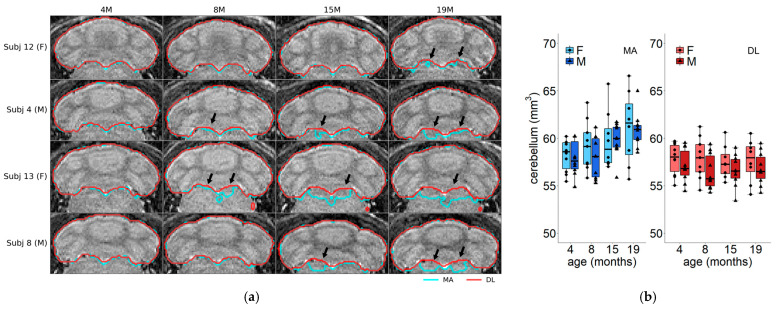
(**a**) Comparison between multi-atlas (blue) and deep learning (red) segmentation of the cerebellum. Representative slices from four subjects (rows) are shown at four time points (columns). Arrows indicate major differences between the two methods. (**b**) Variation in cerebellum volume with age. Dots represent the single-subject values. Group color code: female and multi-atlas (light blue), male and multi-atlas (blue), female and deep learning (light red) and male and deep learning (red). Abbreviations: F = female, M = male, MA = multi-atlas segmentation, DL = deep learning segmentation.

Experimental data are reported in Figure 3 (except for cerebellum data, which are reported in Figure 4) for each region; they have been divided by segmentation method and further grouped by sex.

Our preliminary results show that the whole-brain volume significantly increased by about 3% of the initial size with age (i.e., from 4 to 19 months) (*p* = 0.0003). Therefore, to exclude results solely driven by increases in body and brain mass, we included the whole-brain volume as a covariate in the LMM analysis.

LMM analysis shows that the two methods produce significantly different volumes, for all regions except the white matter, at 4 months of age (model intercept). There is a significant difference in aging trajectory (slope) between the two methods in four regions (cerebellum, cortex, striatum and white matter). All details are summarized in Table 1. For a complete report of the statistical analysis, please refer to the data repository.

When a linear model was applied to compare the two groups, a change in the intercept was observed, without a change in the slope, which suggests that the two groups follow parallel trends. This means that changing the segmentation method introduces a constant offset at every age. Such a constant offset allows the comparison between the two groups to be preserved (treated vs. untreated; pathological vs. control) at every time point (Supplementary Figure S3). This means that, unless we can prove that one of the methods is closer to the real segmentation, we do not know which of the two is more accurate; however, we can trust their results in a between-groups comparison.

Special attention should be paid to regions where both the intercept and the slope significantly differ in the linear mixed model (LMM) analysis—specifically the striatum, white matter, cortex and the cerebellum. In this case, age trajectories are not parallel anymore, and between-groups comparisons cannot be performed at different ages.

For three of these four brain regions (cortex, striatum and white matter), both methods result in slopes with the same direction: negative for the striatum and cortex and positive for white matter.

On the contrary, for the cerebellum (Figure 4), according to the multi-atlas technique, the volume increases with age for both males and females (slope = 0.07477, *p* = 0.0012), but the DL method detects a reducing effect (slope = −0.1362, *p* < 0.0001).

To determine the origin of this remarkable difference, the region contours obtained via the two methods were overlaid on the structural MRI image. Figure 4a shows a representative slice of four subjects (two males and two females) along time. These plots reveal that at 4 months of age, the two methods are in good agreement, but they diverge as the subjects grow older. From 8 months onwards, the multi-atlas method estimates bigger volumes than the DL method (Figure 4b). A comparison with the histological mouse atlases show that the DL method better approximates the mouse cerebellum shape at the selected brain coordinates (Allen Mouse Brain Atlas, https://atlas.brain-map.org, accessed on 30 May 2025). It is worth noting that mismatched segmentations seem to be located in well-defined sub-regions. For example, in the case of the cerebellum, mismatched areas increased in size with age but were approximately in the same location (arrows in Figure 4a). This also holds for the olfactory bulbs (Supplementary Figure S1), where the slope is not affected by method. Such a systematic difference may be an indication that both methods deliver results close to the real segmentation, but with a different degree of accuracy. We note that such a difference still permits an accurate comparison between two experimental groups (e.g., healthy vs. unhealthy, treated vs. control).

### 3.3. Effect of Age on Brain Volume

As already mentioned in the previous section, we observed a significant increase in total brain volume over a lifetime for both males and females. It should be noted that, when sex is added as a covariate, the LMM detects an interaction between sex and age (*p* = 0.0014), which indicates that females have a higher brain growth rate compared to males (Figure 3a and Table 1).

Along with the whole brain, other regions show significant progressive growth (positive slope; significant age effect), namely the ventricles, white matter, thalamus and hypothalamus, and olfactory bulbs (Figure 3b–e). Other regions undergo a reduction (negative slope), i.e., the cortex, striatum, and amygdala (Figure 3f–h), while the hippocampus remains unchanged (Figure 3i).

For both the ventricles (Figure 3b) and the thalamic/subthalamic regions (Figure 3d), the age*method model reveals a significant age effect (*p* < 0.0001). Both methods result in a significant positive slope (*p* < 0.0001). By adding sex as a covariate in the model, we found that both sexes are characterized by a similar, significant enlargement during aging.

A similar trend is observed for the white matter region (Figure 3c): a significant age effect (*p* < 0.0001) is found, with a significant positive slope (*p* < 0.0001) for both methods. Moreover, the age*method model also detects a significant interaction (*p* = 0.0113). This highlights that both methods detect a significant enlargement, but there is a significant difference in the value of their estimates. Adding sex as a covariate does not alter these results.

Finally, the olfactory bulbs were also found to increase with age (Figure 3e). The age*method model detects a significant enlargement (*p* < 0.0001) of the olfactory bulbs over time. In this case, adding sex as a covariate confirms the previous findings; an additional effect of sex on the intercept (*p* = 0.001) is observed, with a significant interaction with method (*p* = 0.0029) and with age (*p* < 0.0001). When comparing the mean volume at 4 months, we found that males’ olfactory bulbs are larger those of females. However, when analyzing their change over time, we found that the rate of enlargement in males is lower during the lifespan (*p* < 0.0001). The overall effect is that after 8 months of age, there is no significant difference between males and females.

Despite an overall enlargement of the brain, the cortex, striatum and amygdala were observed to shrink during aging; this is an example of a sexual dimorphism in mice.

The cortex volume (Figure 3f) significantly decreased with age (*p* < 0.0001), with the model also detecting a slightly significant interaction between method and age (*p* = 0.0310). When the sex variable is added to the model, we find that it significantly affects the volume at 4 months (*p* < 0.0001). Both methods highlight a significant reduction in volume with increasing age. Females have larger cortices at 4 months with respect to males, and this remains the case during their lifetime.

Our results for the striatum (Figure 3g) are, to some extent, more difficult to interpret. The age*method model revealed a significant effect of age (*p* < 0.0001) and a significant method*age interaction (*p* = 0.007). As with the cortex, both methods are in agreement and show a reduction in volume with age. Both slopes go in the same direction, but significantly differ in value (*p* = 0.007). When the sex variable is added to the model, the previous findings are confirmed; i.e., sex significantly affects the intercept of the model (*p* = 0.0006) and interacts significantly with method (*p* = 0.0016). The triple age*method*sex interaction (*p* = 0.0417) was also significant. Both model estimates show that both males and females undergo a significant decrease as a function of age. Moreover, the multi-atlas technique reveals a difference (females > males) starting from 4 months that increases with age. DL segmentation, on the other hand, detects differences between males and females later, at more than 8 months of age. Such results highlight that the multi-atlas technique detects a sexual dimorphism as early as 4 months of age, but the DL method results indicate that the difference between sexes gradually appears during aging.

For the amygdala, the LMM reveals a significant effect of age (*p* = 0.037) in terms of a progressive reduction in the region, as well as an effect of sex on the volume at 4 months (*p* = 0.0058) and a sex*method interaction (*p* = 0.0161). A significant negative slope (MA: *p* = 0.024; DL: *p* = 0.0037) is observed in male subjects, but not in females, with both methods.

Finally, the hippocampus undergoes a significant increase in volume, but the significance level is not reached when using the whole-brain volume as a covariate.

## 4. Discussion

This study has shown that, provided a reasonably small number of pre-segmented MRI brain scans, a deep learning network can be trained to segment unseen brains with a similar accuracy to more established methods, such as the multi-atlas technique, but in a fraction of the duration. More precisely, by using a deep learning approach, a user can segment a brain in approximately 13 to 124 s, depending on whether they have access to a GPU card. The main disadvantage of this segmentation method is the relatively small number of regions and the specific nature of their compositions: it is likely that researchers will be more interested in larger regions or, alternatively, more granular regions, to address specific research questions. Such a need can only be met by retraining the model via customized regrouping of brain regions. A voxel-based approach can also be employed if one needs to estimate local effects of age or sex on cortical aging in mice.

Another important point that arises when comparing the two methods is deciding which one produces a more reliable segmentation result. Manual segmentation is still considered the “gold standard”, although it has long been known for being subject to bias by intra (same operator, different time)- and inter (different operator)-operator variability [26,27]. Mostly, this variability arises from the ambiguity of structural boundaries when the resolution and contrast-to-noise ratio are low. In animal models, in which the in vivo isotropic MRI resolution rarely reaches more than 0.1 mm, partial volume effects can make the segmentation of some brain regions difficult (e.g., in the striatum). Multiple atlases for murine brain segmentation have been constructed over the last few years and are publicly accessible [25,28,29], but a consensus has not been reached among the scientific community due to many factors, including the different acquisition contrasts and data sources (in vivo or ex vivo), the lack of a one-to-one label correspondence between atlases, and the high homogeneity of chosen populations (same strain, same age, and usually males). All these factors also contribute to the difficulty in comparing studies. Here, for example, when we observed the cerebellum or olfactory bulbs, the best multi-atlas segmentation was observed for male mice at 4 months of age, which is the very population we used to create the multi-atlas. On the other hand, generating valid templates for each subgroup is time-consuming, laborious, and likely to introduce biases. Therefore, even if this comparison was restricted to a single mouse strain with a relatively small number of brain labels, our data suggest that DL is more capable of managing the differences that strain, sex and age may introduce.

Despite the limitations mentioned above, most of the biological findings of this work are confirmed by both segmentation methods, which highlight some important features about the normal aging of C57Bl/6J mice—the most common mouse strain used in preclinical research.

Over a long time, the scientific community has demonstrated important functional and morphological changes in the human brain during healthy aging, hence the need to better understand what is considered normal. The aim of studying brain normal aging in animal models is twofold: to distinguish pathological aging and to determine if some brain regions better mimic human features than others. A few previous studies have investigated normal aging in rats [28,29] and mice [30,31]. However, all these studies have concentrated their efforts on male subjects and have a cross-sectional design; moreover, mice characterization was performed post mortem. Thus, our study is, to our knowledge, the first longitudinal neuroimaging study that investigates, at the single-subject level, the effect of aging on a mouse model from a healthy population.

The most consistent finding in the mouse and human literature regards the aging trajectory of the cortex. Many studies that have compared young adult and aged mice within the healthy population have found progressive cortical thinning [31,32,33,34]. The same phenomenon was observed in humans [35,36], suggesting that cortical thinning might be a biologically relevant feature common in mammals, the investigation of which may help to characterize normal and pathological aging.

In this study, we also investigated the influence of sex on longitudinally observed changes in the cerebral structure. The importance in brain sexual dimorphism has grown in the last few years due to the diverse pathological manifestations and incidence of neurodegenerative diseases in men and women, and has driven new gender-oriented therapeutic strategies [37]. Thus, it is becoming important at the experimental level to differentiate biological observations by sex. If cortical thinning has been previously detected in animal models, the role of sex is still not clear. In fact, some studies have detected a more pronounced age-related reduction in cortical structure volume for males than females [38], but others did not [35,39]. Here, we observed a more complex scenario: females have a significantly larger cortex at 4 months of age and their rate of cortex shrinkage is lower, suggesting a better preservation of cortical structures.

Another brain region with similar development in humans is the striatum. In our study, we found that the striatum significantly decreases in size with age, as previously suggested by other studies in rats [28,40] and in a cross-sectional study in mice [31]. Correspondently, in humans, it has also been found to shrink with age, even in healthy subjects [41,42]. When sex was introduced as a covariate, our results show that males are characterized by a faster decline; in humans, it has also been found that striatal loss of volume is accelerated in males [42].

Ventricles expectedly enlarged at the expense of surrounding regions, and they were also observed to do so in other studies in both humans [36] and rodents [28,30,32].

Despite such remarkable similarities between humans and mice, other brain areas exhibit different developmental patterns. Our results show that the whole brain of mice still significantly enlarges from adulthood to old age (around 3–4%), as suggested by two more studies which compared size changes between young and old mice [32,34]. The human brain, on the contrary, shows a progressive general atrophy in adulthood [36]; it reaches its maximum volume at around 18 years old and then starts to slowly decrease [35].

Like the whole brain, white matter tracts in mice are characterized by a progressive enlargement up to old age. In humans, the relationship between white matter volume and age follows a bell-shaped trajectory, with an increase until the 30s and a progressive decline after the 50s [35].

Such a similarity in the age trajectory of some brain areas, and the fact that other trajectories are consistent within, but not across, species, may help in understanding the relation between brain size and lifespan [43] and guide more focused studies in rodent models.

In conclusion, we propose a deep learning tool for segmentation of the murine brain. In comparison with an atlas-based approach, DL is faster and more robust to ambiguity when the group under study is different from the reference atlas.

Moreover, our segmentation methods, applied to a cohort of healthy, aging C57Bl6/J mice, have highlighted that rodent brains exhibit volume modification with age. More importantly, some key brain regions are characterized by sexual dimorphism: investigating these regions may help understand the role of sex in brain maturation and neurological pathologies in the absence of social and cultural differences.

## Figures and Tables

**Table 1:** Effect of method (the multi-atlas technique vs. deep learning) on LMM estimates.

Brain Region	Intercept ^1^	Slope ^2^		
	Test for Differences by Method	Sign MA	Sign DL	Test for Differences by Method
whole brain	(**)	+	+	(ns)
cortex	(****)	−	−	(*)
striatum	(****)	−	−	(**)
white matter	ns	+	+	(*)
cerebellum	(****)	+	−	(****)
hippocampus	(*)			(ns)
amygdala	(****)	−	−	(ns)
olfactory bulbs	(****)	+	+	(ns)
thalamus and hypothalamus	(****)	+	+	(ns)
ventricles	(****)	+	+	(ns)

^1^ Estimated volume at 4 months of age. ^2^ MA = multi-atlas, DL = deep learning, + = positive effect of age, − = negative effect of age. ns = not significant, * *p* < 0.05, ** *p* < 0.01, **** *p* < 0.0001.

## Data Availability

The data presented in this study are openly available in NeuGRID at www.neugrid2.eu (accessed on 30 May 2025) and Zenodo at https://doi.org/10.5281/zenodo.17130659.

## Supplementary Materials

The following supporting information can be downloaded at: https://www.mdpi.com/article/10.3390/jimaging11110418/s1, Figure S1: Comparison between multi-atlas and deep learning segmentation of the olfactory bulbs; Figure S2: The linear mixed model; Figure S3: Sketch of the contrasting effects of method on the intercept and slope of the model; Table S1: Ex vivo atlas remapping.

## Abbreviations

The following abbreviations are used in this manuscript:

MRI	Magnetic Resonance Imaging
DL	Deep Learning
MA	Multi Atlas
LMM	Linear Mixed Model
DSC	Dice Similarity Coefficient
CPU	Central Processing Unit
GPU	Graphic Processing Unit
