# Supplementary material for: Deep Learning and Atlas-Based MRI Segmentation Enable Longitudinal Characterization of Healthy Mouse Brain
Source: J Imaging. 2025 Nov 19;11(11):418. doi: 10.3390/jimaging11110418 (PMC12653442; doi:10.3390/jimaging11110418)
Supplement: Supplementary file 1 [file jimaging-11-00418-s001.zip › Micotti_supplementary_figures.pdf]

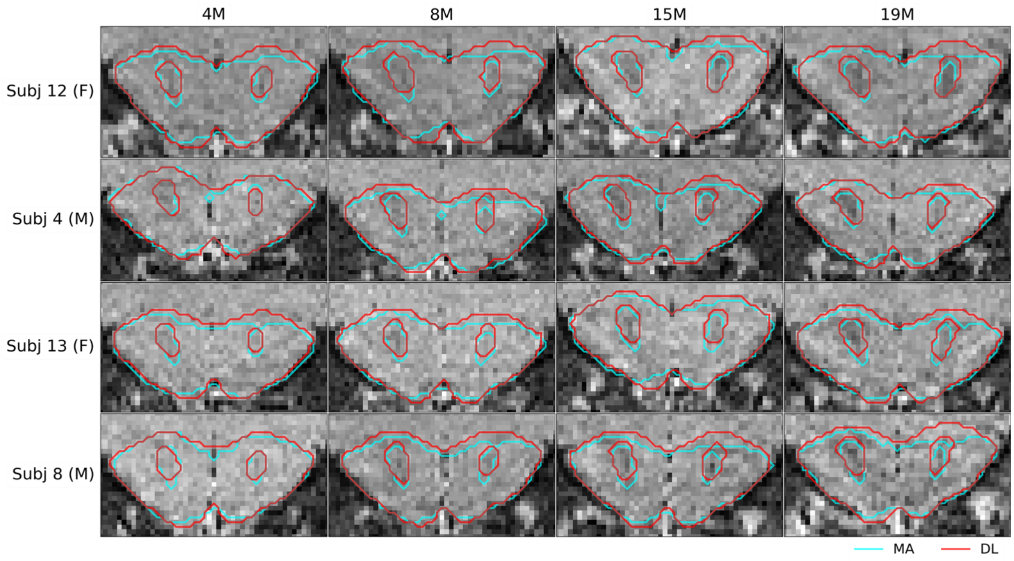

Supplementary Figure S1. Comparison between multi-atlas (blue contours) and deep learning (red contours) segmentation of the olfactory bulbs. Representative slices from four subjects (rows) are shown at four time points (columns). Arrows indicate major differences between the two methods.

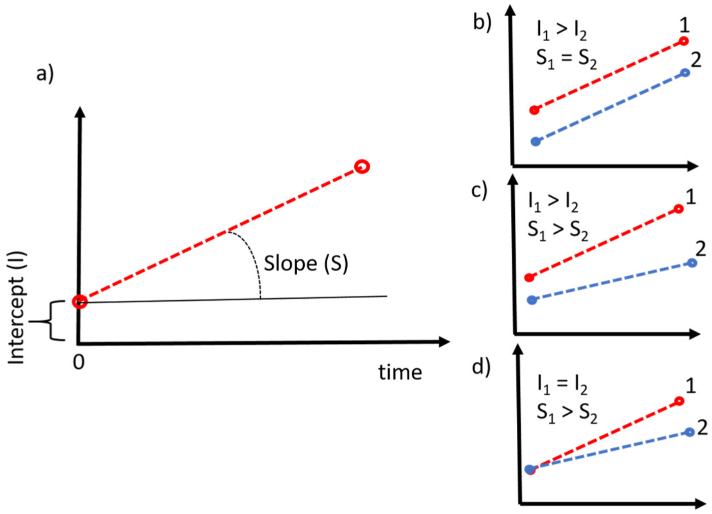

Supplementary Figure S2. a) Sketch of the outputs of LMM: intercept (I) and slope (S). Panel b) c) and d) show three possible scenarios for the ageing trajectory when comparing group 1 and group 2.

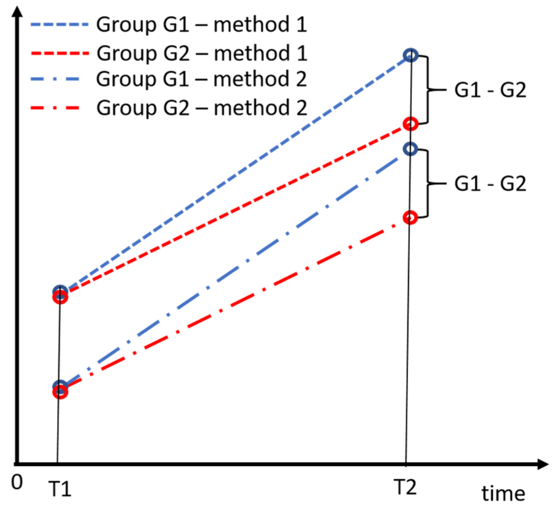

Supplementary Figure S3. Sketch of the effect of method on the intercept of the model but not on slope. At T1(T2), absolute measure of G1(G2) is different between methods but the effect of G1 vs G2 remains unchanged.
