# Supplementary material for: Deep Learning and Atlas-Based MRI Segmentation Enable Longitudinal Characterization of Healthy Mouse Brain
Source: J Imaging. 2025 Nov 19;11(11):418. doi: 10.3390/jimaging11110418 (PMC12653442; doi:10.3390/jimaging11110418)
Supplement: Supplementary file 1 [file jimaging-11-00418-s001.zip › Micotti_Table_S1.pdf]

Supplementary Table S1. Ex-vivo atlas remapping.

Region of interest	Structure (Dorr et al., 2008)	Label number (Dorr et al., 2008)
amygdala	amygdala	151, 51
cerebellum	arbor vita of cerebellum	47, 247
cerebellum	cerebellar cortex	2, 202
cerebellum	cerebellar peduncle: inferior	223, 123
cerebellum	cerebellar peduncle: middle	245, 45
cerebellum	cerebellar peduncle: superior	222, 242
cortex	cerebral cortex: entorhinal cortex	230, 209
cortex	cerebral cortex: frontal lobe	190, 64
cortex	cerebral cortex: occipital lobe	164, 130
cortex	cerebral cortex: parieto-temporal lobe	180, 181
hippocampus	dentate gyrus of hippocampus	66, 16
hippocampus	hippocampus	106, 6
hippocampus	pre-para subiculum	131, 133
hippocampus	stratum granulosum of hippocampus	63, 13
olfactory bulbs	lateral olfactory tract	102, 101
olfactory bulbs	olfactory bulbs	105, 5
olfactory bulbs	olfactory tubercle	145, 95
olfactory bulbs	subependymal zone / rhinocele	140, 240
striatum	fundus of striatum	154, 54
striatum	nucleus accumbens	155, 55
striatum	striatum	17, 7
thalamus and hypothalamus	hypothalamus	150, 250
thalamus and hypothalamus	mammillary bodies	61, 161
thalamus and hypothalamus	mammillothalamic tract	212, 210
thalamus and hypothalamus	thalamus	4, 204
ventricles	cerebral aqueduct	119
ventricles	fourth ventricle	118
ventricles	lateral ventricle	77, 57
ventricles	third ventricle	146
white matter	anterior commissure: pars anterior	215, 115
white matter	anterior commissure: pars posterior	103, 23
white matter	cerebral peduncle	14, 114
white matter	corpus callosum	68, 8
white matter	fasciculus retroflexus	125, 25
white matter	fimbria	11, 211
white matter	fornix	22, 122
white matter	internal capsule	12, 112
white matter	medial lemniscus/medial longitudinal fasciculus	120, 20
white matter	optic tract	116, 216
white matter	posterior commissure	100
white matter	stria medullaris	205, 225
white matter	stria terminalis	159, 59
other	basal forebrain	152, 52
other	bed nucleus of stria terminalis	76, 176

other	colliculus: inferior	43, 143
other	colliculus: superior	109, 9
other	corticospinal tract/pyramids	18, 218
other	cuneate nucleus	168, 166
other	facial nerve (cranial nerve 7)	219, 19
other	globus pallidus	144, 44
other	habenular commissure	199, 99
other	inferior olivary complex	213, 113
other	interpeduncular nucleus	157
other	lateral septum	107, 207
other	medial septum	153, 53
other	medulla	174
other	midbrain	194
other	periaqueductal grey	10
other	pons	187
other	pontine nucleus	185, 85
other	superior olivary complex	214, 124
other	ventral tegmental decussation	156
